# Neuropsychological and clinical variables associated with cognitive trajectories in patients with Alzheimer's disease

**DOI:** 10.3389/fnagi.2025.1565006

**Published:** 2025-05-27

**Authors:** Marianna Riello, Monica Moroni, Stefano Bovo, Flavio Ragni, Manuela Buganza, Raffaella Di Giacopo, Marco Chierici, Lorenzo Gios, Matteo Pardini, Federico Massa, Monica Dallabona, Elisa Vanzetta, Cristina Campi, Michele Piana, Sara Garbarino, Manuela Marenco, Venet Osmani, Giuseppe Jurman, Antonio Uccelli, Bruno Giometto, NeuroArtP3 Network, Filippo Gerli

**Affiliations:** ^1^Neurology Unit, Provincial Health Services of Trento, Trento, Italy; ^2^Data Science for Health Unit, Fondazione Bruno Kessler, Trento, Italy; ^3^TrentinoSalute4.0, Trento, Italy; ^4^IRCCS Ospedale Policlinico San Martino, Genoa, Italy; ^5^Department of Neuroscience, Rehabilitation, Ophthalmology, Genetics, Maternal and Child Health (DINOGMI) Università di Genova, Genoa, Italy; ^6^Department of Mental Health, Division of Psychology, Provincial Health Services of Trento, Trento, Italy; ^7^CISMed, University of Trento, Trento, Italy

**Keywords:** Alzheimer dementia, mild cognitive impairment, MMSE, machine learning, random forest, SHAP analysis

## Abstract

**Background:**

The NeuroArtP3 (NET-2018-12366666) is a multicenter study funded by the Italian Ministry of Health. The aim of the project is to identify the prognostic trajectories of Alzheimer's disease (AD) through the application of artificial intelligence (AI). Only a few AI studies investigated the clinical variables associated with cognitive worsening in AD. We used Mini Mental State Examination (MMSE) scores as outcome to identify the factors associated with cognitive decline at follow up.

**Methods:**

A sample of *N* = 126 patients diagnosed with AD (MMSE >19) were followed during 3 years in 4 time-points: T0 for the baseline and T1, T2 and T3 for the years of follow-ups. Variables of interest included demographics: age, gender, education, occupation; measures of functional ability: Activities of Daily Living (ADLs) and Instrumental (IADLs); clinical variables: presence or absence of comorbidity with other pathologies, severity of dementia (Clinical Dementia Rating Scale), behavioral symptoms; and the equivalent scores (ES) of cognitive tests. Logistic regression, random forest and gradient boosting were applied on the baseline data to estimate the MMSE scores (decline of at least >3 points) measured at T3. Patients were divided into multiple splits using different model derivation (training) and validation (test) proportions, and the optimization of the models was carried out through cross validation on the derivation subset only. The models predictive capabilities (balanced accuracy, AUC, AUPCR, F1 score and MCC) were computed on the validation set only. To ensure the robustness of the results, the optimization was repeated 10 times. A SHAP-type analysis was carried out to identify the predictive power of individual variables.

**Results:**

The model predicted MMSE outcome at T3 with a mean AUC of 0.643. Model interpretability analysis revealed that the global cognitive state progression in AD patients is associated with: low spatial memory (Corsi block-tapping), verbal episodic long-term memory (Babcock's story recall) and working memory (Stroop Color) performances, the presence of hypertension, the absence of hypercholesterolemia, and functional skills inabilities at the IADL scores at baseline.

**Conclusion:**

This is the first AI study to predict cognitive trajectories of AD patients using routinely collected clinical data, while at the same time providing explainability of factors contributing to these trajectories. Also, our study used the results of single cognitive tests as a measure of specific cognitive functions allowing for a finer-grained analysis of risk factors with respect to the other studies that have principally used aggregated scores obtained by short neuropsychological batteries. The outcomes of this work can aid prognostic interpretation of the clinical and cognitive variables associated with the initial phase of the disease towards personalized therapies.

## 1 Introduction

Alzheimer's Dementia (AD) represents a major cause of disability and mortality for 44 million people worldwide and this number is expected to triple as the population ages by 2050 (Lane et al., 2018). Although AD is recognized by the World Health Organization as a global public health priority, the first pharmacological treatments have not yet been accepted and introduced into guidelines by all the competent agencies in the world. Also, despite the monoclonal antibodies, used to treat amyloid accumulation in early AD, have recently received approval from American Food and Drug Administration (US-FDA), the relationship between cognitive benefit and side effects is still controversial (Cummings et al., 2024). For these reasons the identification of factors which might influence the cognitive trajectories of the disease could be crucial for a preventive therapy sought for AD.

Clinical consensus is consistent in considering AD a syndrome characterized by a continuum of clinical and biological phenomena rather than three distinct clinically defined entities known as preclinical AD, mild cognitive impairment (MCI) and dementia (McKhann et al., 2011; Dubois et al., 2016; Jack Jr. et al., 2018). More specifically, patients progress from normal cognition to MCI due to AD, and subsequently experience an increase in the severity of AD dementia (mild, moderate, and severe) (Sperling et al., 2011). However, accurate prediction of AD trajectories over time still remains a challenging task due to the complicated characteristics of the disease progression. Within this continuous process, many factors might present an additive effect on cognition contributing to AD cognitive worsening, but the interplay between these variables is still not well understood. For example, despite the extensive evidence showing the crucial role of amyloid in driving cognitive decline, this finding does not fully explain the complexity of late-life cognitive deterioration (Jagust et al., 2023). In fact, due to the different combinations between biomarker profiles and cognitive stages in which AD occurs, it is still unclear whether the cognitive deficit is attributable to AD alone or to other potential comorbidities (Jack Jr. et al., 2018). These includes early life risk factors, such as years of education (Xu et al., 2016), and some midlife and later life components, including brain injury (Li et al., 2017), hypertension (McGrath et al., 2017), diabetes (Yen et al., 2022), depression (Singh-Manoux et al., 2017; Wang et al., 2018) and cerebrovascular diseases (Wang et al., 2018, Xia et al., 2020, Rundek et al., 2023; see also the 14 risk factors model by Livingston et al., 2024). As the disease progresses, individuals with pathological evidence of AD, but cognitively normal, named “preclinical AD” patients (Morris et al., 2009; Vos et al., 2013), might suffer from cognitive decline that is detectable by sensitive neuropsychological measures (Albert et al., 2011). In particular, cognitive tasks assessing memory deficits as semantic, episodic memory and executive functions, might be sensitive in predicting future clinical AD (Amieva et al., 2008; for reviews see review: Twamley et al., 2006; Salmon, 2012; Mortamais et al., 2017). According to these assumptions, it might be of great importance to identify factors influencing the worsening of the disease and on which we can act promptly.

Artificial intelligence (AI) models might play a significant role in this context, due to their ability in leveraging massive amounts of data and uncovering intricate patterns and relationships that might be missed by traditional statistical methods. Several studies applied AI models on patients data to highlight the role of different factors in AD diagnosis and progression, focusing mainly on neuroimaging data, such as structural Magnetic Resonance Imaging (MRI) (e.g., Zeng et al., 2018; Lahmiri and Shmuel, 2019), functional MRI (fMRI) (e.g. Sheng et al., 2019), Positron Emission Tomography (PET) (e.g. Peng et al., 2019) and Single-photon Emission Computerized Tomography (SPECT) (e.g. Segovia et al., 2010; for a review see Tanveer et al., 2020).

In recent literature, most of the studies aimed at modeling AD progression used data based on standardized publicly available multimodal dataset (Kumar et al., 2021). Several recent studies applied AI to electronic health records (EHRs) to predict AD progression showing the predictive value of neuropsychological data on cognitive decline (Zhu et al., 2016; Fisher et al., 2019; Dansson et al., 2021). However, despite the use of big dataset and highly accurate model performance, these studies did not explore the contribution of individual cognitive tests to the diagnostic outcome. For example, the brief neuropsychological assessment named Alzheimer's Disease Assessment Scale (ADAS-Cog) (Rosen et al., 1984) included in several EHRs might not be as predictive as the performance at individual cognitive tests used in in-depth cognitive evaluation. In general, for precision medicine, it may be appropriate when the screening tests, such as the Mini Mental State Examination (MMSE) (Folstein et al., 1975), are followed by a wider comprehensive battery of tests, administered by expert neuropsychologists and measuring specific cognitive abilities (Riello et al., 2021) as described by the European diagnostic workflow (Frisoni et al., 2024). The aim of this study is to identify which factors are associated with worsening MMSE scores at 3-year follow-up, represented by demographic, clinical, functional and cognitive variables. The MMSE is one of the most widely used screening tests in clinical practice to assess the global cognitive functioning, is used as an indicator of dementia onset (Arevalo-Rodriguez et al., 2015; Riello et al., 2021) and can provide useful data for the rate progression of the cognitive decline (Chow et al., 2006). We expect these results might be useful for the realization of a personalized approach aiming to reduce cost, increase effectiveness of disease treatment and optimize care.

## 2 Materials and methods

### 2.1 Data Collection

This is a multi-center, retrospective, observational study involving patients admitted to the local healthcare trust - Azienda Provinciale per i Servizi Sanitari (APSS) of Trento (Italy) and the IRCCS (Scientific Institute for Research, Hospitalization, and Healthcare) San Martino Hospital of Genoa (Italy). Data selected for this study was collected as part of the standard routine practice. The centers collected data from patients diagnosed with AD at MCI or early dementia stages, in accordance with established diagnostic criteria (Albert et al., 2011; McKhann et al., 2011), providing longitudinal follow-up from the diagnosis/recruitment (baseline). Data were therefore collected at different timepoints, namely at baseline visit (T0) and at three subsequent follow-ups (FU) (at 12, 24, and 36 months, T1-T2-T3). Inclusion criteria were: (1) MMSE >19; (2) Patients who received a diagnosis of AD between May 2006 and August 2020. All AD patients met the criteria for probable AD with at least intermediate likelihood based on (Albert et al., 2011; McKhann et al., 2011). In details, our patients (N = 126) met the core clinical criteria (decline from previous level of functioning, gradual onset over months, evidence of lower performance in one or more cognitive domains with amnestic and non-amnestic presentations, not explained by delirium or other major neurodegenerative or psychiatric disorders) and the presence of neuronal injury imaging data (data from structural MRI and/or from [18F]FDG PET). Furthemore, 62.7% of patients was also considered at high likelihood of AD since they presented positive amyloid biomarkers from Amyloid-PET with specific tracers and/or cerebrospinal fluid (CSF) assessment of amyloid isoforms (Aβ42/Aβ40 ratio), also in accordance with the proposed AT(N) research framework (Jack Jr. et al., 2018). Variables of interest were decided on the basis of the retrospective available data in the two centers. Data collected were grouped into the following categories: (1) Demographic variables including: age, education, gender and working position; (2) Clinical features: duration of the disease, family history for a group of diseases, presence or absence of habits (alcohol and smoking self reported habits) in the past and at diagnosis, presence or absence of comorbidities (hypertension, hypercholesterolemia, head trauma, diabetes, heart disease, liver disease, thyroid disease, tumors, cerebrovascular disease) assessed by specialists according to the national guide-lines, severity of dementia at the Clinical Dementia Rating Scale (CDR) (Morris, 1993), presence or absence of motor aspects (falls, dysphagia, parkinsonism as apraxia or pyramidal signs), assessed by the neurologists involved in the study through the neurological examination, presence or absence of behavioral symptoms (depression, apathy, hallucinations/delusions, aggression, disinhibition/inadequate behavior, specific sleep-wake rhythm disorders) collected from the administration of the Neuropsychiatric Inventory (NPI; Cummings, 1997), and pharmacological therapy; (3) measures of functional daily abilities evaluated by the Activities of Daily Living (ADLs) (Katz et al., 1963) and Instrumental activities (IADLs; Lawton, 1969) and, (4) Cognitive data: in the form of equivalent scores (ES) of the neuropsychological assessment covering the following domains: general cognitive functioning measured by the MMSE and the Montreal Cognitive Assessment (MoCA; Nasreddine et al., 2005); memory functions examined by the Forward Digit Span and the Corsi block-tapping test (Monaco et al., 2013), the Rey Auditory Verbal Learning Test (RAVLT; Carlesimo et al., 1996) and the Babcock's Story Recall Test (BSRT; Novelli et al., 1986), language assessed by the semantic verbal fluency test (Novelli et al., 1986), attention investigated by the Trail Making test A-B (Amodio et al., 2002), executive functions measured by the phonological verbal fluency test (Carlesimo et al., 1995) and the Stroop Color and Word Test (SCWT; Barbarotto et al., 1998) and visuospatial abilities assessed by the Clock Drawing Test (CDR; Caffarra et al., 2011). For additional information about the collected variables, see Supplementary Table S1.

### 2.2 Data processing

The outcome variable, namely the presence of global cognitive impairment, was defined as a decrease of at least 3 points at the MMSE test score measured at the 3 years follow-up (Zhu et al., 2016).

Before being parsed by predictive models, the collected variables included in this study underwent a common preprocessing phase. The first step was to remove variables with more than 30% of missing values, no variability or with less than 10% of samples in the minority class. Features with a high proportion of missing values, no variability or highly unbalanced, in fact, may not provide reliable information, potentially introducing noise in the training process and limiting the overall performance of machine learning models. For the remaining predictors, missing values were imputed with the median for numerical variables, while for ordinal and categorical variables the most frequent value was used. ADL and IADL scores were considered as fraction between the number of preserved and total number of tested activities. According to this definition, ADL and IADL scores equal to 1 denote full independence, while values smaller than 1 denote impairment in some functionalities with smaller values denoting greater impairment. Before training the models, dataset variables were normalized using a quantile transformer, a min-max scaler, an ordinal encoder and one hot encoder for numerical, binary, ordinal and categorical features respectively.

### 2.3 Model selection

To ensure optimal model selection, the full dataset was divided into a derivation (training) and validation (test) set. The training set was used to fit and optimize models, while the test set was employed to evaluate models' performance.

Since the train-test split selection might influence results due to the intrinsic variability between different subsets, multiple dataset partitions were used during the training process. This approach enabled the assessment of the robustness and stability of the tested algorithms: in fact, a model that performs consistently well across different splits is more likely to be reliable and generalizable. Therefore, starting from the whole dataset, four train-test splits were created using different proportions (60%–40%, 70%–30%, 80%–20%, 90%–10%).

The performances of three machine learning models, namely Logistic regression (LR), Random Forest (RF), and Gradient Boost (GB) were compared. LR is a classic supervised machine learning algorithm, mainly used for baseline binary classification problems where the goal is to predict the probability that an instance belongs to a given class or not. The logistic function is used to describe the relationship between the independent variables and the selected outcome. For each input, the model computes the probability that a given input belongs to a certain class and then makes a prediction based on a chosen threshold.

RF consists of several independently trained decision trees that work together to provide a single output. A random subset of the data set is used to build each tree to measure a random subset of features in each partition. To make a prediction for a classification task, the algorithm aggregates the results of all trees by voting. The combination of randomness and collaborative decision-making process, reduces the risk of overfitting and provides stable and precise results.

GB is a powerful machine learning algorithm for classification and regression tasks. Similarly to RF, this is a method that combines the predictions of multiple weak learners to create a single, more accurate strong learner (i.e. ensemble learning).

### 2.4 Parameters optimization

The hyperparameters of each model were optimized by means of a randomized grid search procedure. This begins by defining a set of possible values for each model's hyperparameters. Subsequently, a combination of these values is randomly selected, and the model is trained and evaluated with and without Synthetic Minority Over-sampling Technique (SMOTE; Chawla et al., 2002). SMOTE is a method used to address class imbalance in ML datasets and operates by generating synthetic samples for the minority class, thereby artificially balancing class distribution. This process was repeated 100 times for each model using the Optuna hyperparameter optimization framework (Akiba et al., 2019) in a three-fold stratified cross validation setting, repeated three times. The hyperparameter combination (with or without SMOTE) with the highest Matthews Correlation Coefficient (MCC) score was selected (Chicco and Jurman, 2023). MCC is a performance metric that takes into account both true positives (TP), true negatives (TN), false positives (FP) and false negatives (FN), providing a comprehensive evaluation of the quality of binary classification. This entire process was repeated 10 times for each ML model and train-test split proportions, for a total of 40 grid search procedures for each classifier. To evaluate the predictive performance of the models several metrics were considered and computed on the test set, such as: balanced accuracy, the area under the receiver operator characteristic curve (AUC), the area under the precision-recall curve (AUPRC), the F1 score and the MCC.

### 2.5 AI interpretability

To increase the interpretability of our results, Shapley Additive exPlanations (SHAP) method (Lundberg and Lee, 2017) was applied to the best performing model and train-test partition for each of the 10 iterations, to inspect the predictive power of individual variables. SHAP method increases the interpretability deconstructing each prediction into a sum of individual contributions from each variable, emphasizing their influence both at the instance level and throughout the entire population split considered. For each variable, higher SHAP values suggest a positive contribution to the model's prediction of MMSE decline at T3. Moreover, to understand which feature contributed the most to models' prediction, for each of the ten runs a features importance ranking was performed, by sorting features for decreasing SHAP values., i.e. for decreasing importance. Then, the features present consistently in the first ten positions in at least 60% of the runs were selected as most informative.

## 3 Results

### 3.1 Dataset and data preprocessing

Data collection resulted in a total of 126 patients: 25 patients from the APSS of Trento and 101 patients from the IRCCS Policlinico San Martino Hospital of Genoa.

During the preprocessing step the following variables were discarded: alcohol habit, head trauma, diabetes, liver disease, falls, dysphagia, parkinsonism as apraxia or pyramidal signs, hallucinations/delusions, aggression, disinhibition/inadequate behavior due to the low variability in the sample. At the same time, among the cognitive tests, MoCA, Trail Making test A and B and Clock Drawing were eliminated due to the high number of missing values.

After the dataset preprocessing steps, a total of 28 remaining features were selected for the upcoming model training phase, comprising 4 demographic (gender, age at diagnosis, education and occupation), 12 clinical (including: smoke habit and CDR scores; comorbidities: hypertension, hypercholesterolemia, cardiopathy, thyroidopathy, tumors and cerebrovascular disease; family history of diseases and behavioral symptoms: depression, apathy and sleep disorders), 2 functional variables (ADL, IADL) (see Table 1) and 10 cognitive scores of the neuropsychological battery (for a detailed list see Table 2).

**Table 1:** Demographic and clinical variables at baseline included in the predictive model.

**Category**	**Variables**	**No. (%)**
Demographic characteristics	Female	70 (55.5%)
	Age at diagnosis	71.04 ± 7.10 years
	Education years	10.16 ± 4.37 years
Occupation	Elementary	24 (21.23%)
	Medium-low	30 (26.54%)
	Medium	36 (31.85%)
	Medium-high	23 (20.35%)
	Major	13 (11.50%)
	N/A	13 (11.50%)
Smoking status	Non-smoker	73 (56.34%)
	Previous smoker	42 (33.33%)
	Current smoker	11 (8.73%)
CDR Scores	MCI (0)	101 (80.15%)
	Mild (0.5)	25 (19.84%)
	Moderate (1)	0
Comorbidities	Hypertension	65 (51.58%)
	Hypercholesterolemia	61 (48.41%)
	Cardiopathy	16 (12.69%)
	Thyroidopathy	19 (15.07%)
	Tumors	20 (15.87%)
	Cerebrovascular disease (Fazekas >1)	28 (22.22%)
Family history of diseases	None	66 (52.38%)
	Dementia	50 (39.68%)
	Parkinson	5 (3.96%)
	Multiple sclerosis	1 (0.79%)
	Lateral amyotrophic sclerosis	0
	Cerebral tumors	0
	Psychiatric pathology	0
	Other	6 (4.76%)
Behavioral symptoms	Depression	74 (58.73%)
	Apathy	31 (24.60%)
	No sleep disorders	100 (79.36%)
	Sleep behavior disorder	2 (1.58%)
	Insomnia	23 (20.35%)
Functional abilities	ADLs (6/6)	0.98 ± 0.07
	IADLs (8/8)	0.88 ± 0.18

No., number; SD, standard deviation; CDR, Clinical Dementia Rating Scale; MCI, mild cognitive impairment; ADLs/IADLs, Activities of Daily Living\ Instrumental.

**Table 2:** Neuropsychological variables at baseline included in the AI model.

**Variable**	**ES scores mean (±SD), patients no**.
MMSE	25.91 (±2.65, n=126)
Digit span forward	2.76 (±1.34, n=126)
Corsi block-tapping test forward	1.72 (±1.28, n=125)
Immediate recall (RAVLT)	0.95 (±1.34, n=112)
Delayed recall (RAVLT)	0.76 (±1.24, n=113)
BSRT	0.87 (±1.25, n=112)
Semantic verbal fluency test	2.15 (±1.40, n=120)
Phonological verbal fluency	2.56 (±1.48, n=123)
SCWT: Color	2.21 (±1.32, n=98)
SCWT: Color Word	1.82 (±1.36, n=97)

ES, Equivalent Scores; SD, standard deviation; MMSE, Mini Mental State Examination; RAVL, Rey Auditory Verbal Learning Test; BSRT, Babcock's Story Recall Test; SCWT, Stroop Color Word Test.

### 3.2 Predictive variables

The aim of the current study was also to test the predictive power of different machine learning models (LR, RF and GB) to predict cognitive trajectories in AD patients, beyond highlighting the variables associated with a MMSE worsening at T3.

**Figure 1:**
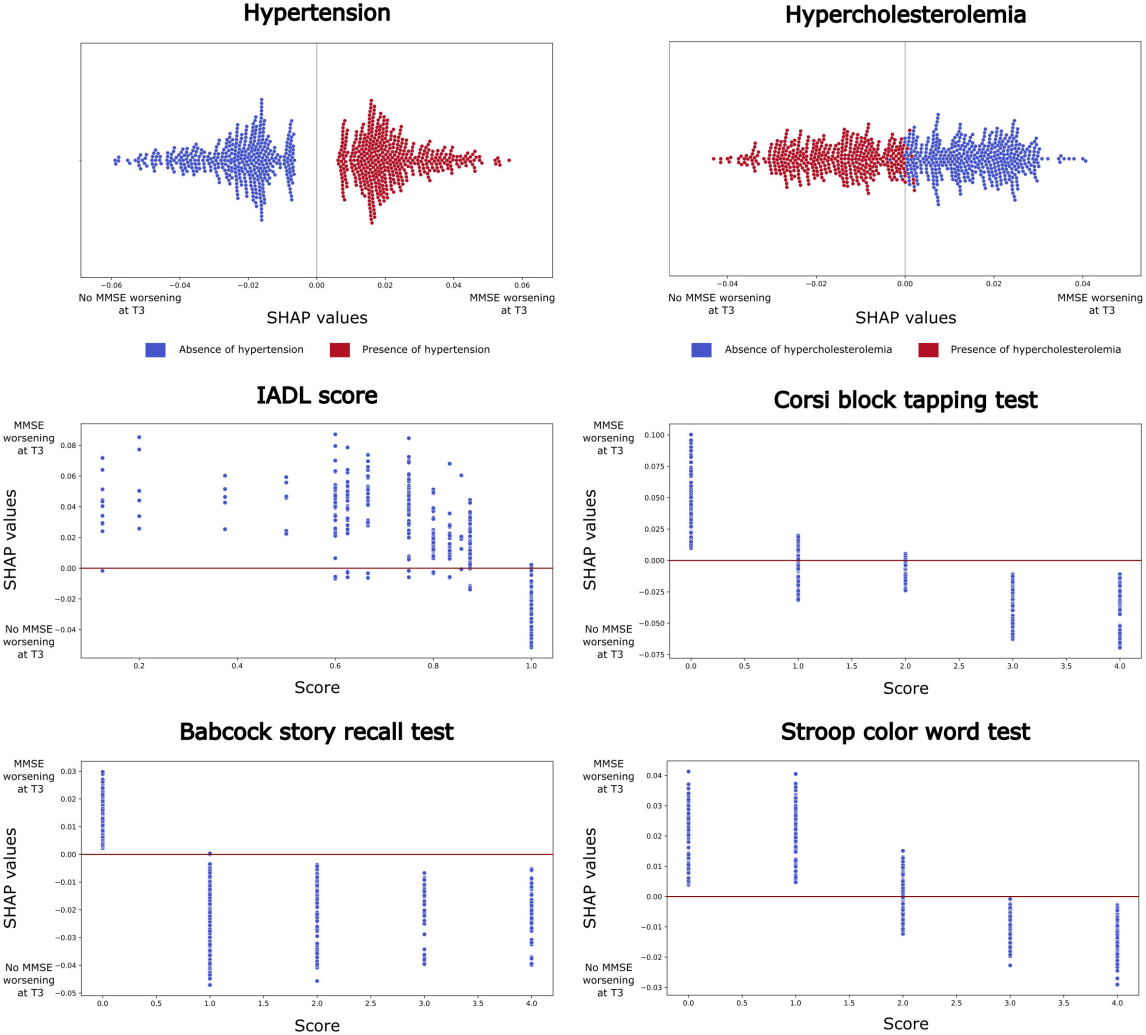
Shapley Additive exPlanations (SHAP) analysis results for the best-performing model (Random Forest, using a 70-30% train-test split). Features that consistently ranked within the top ten across at least 60% of runs are reported. For categorical variables (e.g. hypertension and hypercholesterolemia), beeswarm plots are shown, where each point represents a SHAP value for a feature and an individual observation. Blue points indicate low variable values, while red points indicate high values. For continuous variables (e.g., IADL score, Corsi block tapping test, Babcock story recall, and Stroop color-word test), dependence plots are presented, with each point representing a feature score for an individual participant. Higher SHAP values suggest a positive contribution to the model's prediction of MMSE decline at T3.

The model achieving the best performance and higher stability across all training sessions was RF with a 30% train-test split, with an AUC of 0.643 ± 0.04 (Figure 1). This model was selected due to its small variability across iterations and train-test splits configurations, as shown in Supplementary Table S2, and served as the reference for the interpretability analysis.

**Figure 2:**
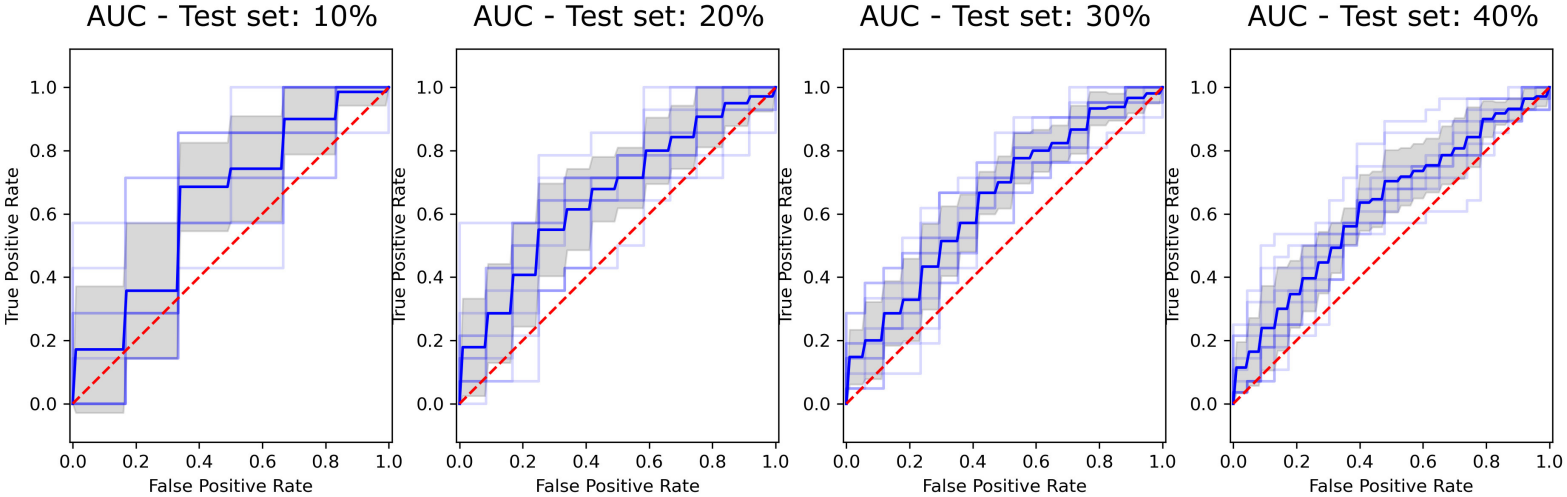
ROC curves showing the performance of the Random Forest (RF) classifier across selected test set sizes (10%, 20%, 30%, and 40%). The True Positive Rate is plotted against the False Positive Rate for each test size, with the Area Under the Curve (AUC) illustrating classifier effectiveness. Blue line represents the mean ROC, the shaded gray area indicates variability across iterations, and the red dashed line corresponds to the line of no-discrimination (AUC = 0.5).

The SHAP analysis, performed to identify the most informative variables consistently present across iterations (>60%), highlighted: 2 clinical, 1 functional and 3 cognitive variables with the highest predictive impact at T3 (Figure 2). Interestingly, the significant predictive variables affected cognitive performance differently. Specifically, the decline of general cognitive abilities measured by MMSE in AD patients was associated with the presence of hypertension and the absence of hypercholesterolemia, also, with impaired functional abilities (IADL < 1, i.e. at least one impaired functionality) and with low performances on the following cognitive tests: the Corsi block-tapping test (ES < 1), the BSRT (ES < 1), and the SCWT (ES < 2).

## 4 Discussion

This study investigated the best AI model to identify which variables, among a combination of demographic, clinical, functional and neuropsychological factors, potentially influence the risk of significant decline of global cognitive functioning, as measured by MMSE in AD patients. Our results demonstrated strong associations between clinical, functional and neuropsychological variables and the MMSE scores at 3 years FU. Patients suffering from hypertension experienced a decline in MMSE scores at T3. In addition, patients with significantly poorer performance on the Corsi block-tapping test, on the BSRT, and, on the SCWT, exhibited a more rapid cognitive deterioration. In contrast, patients with hypercholesterolemia and preserved functional instrumental abilities (IADL=1, i.e. full independence) at baseline did not show cognitive worsening. These findings suggest that some variables may serve as predictors of global cognitive trajectories over time, acting either as a risk or a protective factor for cognitive abilities.

In recent literature, several studies applied AI techniques to investigate which variables influence the worsening of cognitive performance over time in AD patients using clinical data. In particular, Zhu et al., 2016, compared the performance of different machine learning algorithms to predict the decline of MMSE scores at 2-years FU. Their dataset included demographic variables, genetic information and neuropsychological composite measures of memory and executive functions. Later, Fisher et al., 2019 examined which variables, including laboratory tests, clinical, demographic and genetic data, together with cognitive test results, predict cognitive worsening in MCI or AD patients over an 18- month period. More recently, Dansson et al., 2021 applied similar techniques to model patients' cognitive trajectories at 2 and 4 years, by including demographic variables, biochemical-markers (proteins, lipids, hormones), the CSF (Aβ42 and Aβ40) and neuroimaging data (MRI and FDG-PET) as well as a wide range of neuropsychological scores.

Compared to the existing literature, this is the first study predicting cognitive trajectories of AD patients using clinical data only, while at the same time providing explainability of factors contributing to these trajectories. The implemented SHAP analysis, in fact, allows not only to highlight the most important features associated with cognitive decline, but also to unveil how feature values contribute to model prediction. In particular, when applied to neuropsychological tests and IADL, this process might allow the detection of plausible test-specific cut-offs indicative of cognitive decline.

Furthermore, the retrospective dataset considered in the current work provides some crucial advantages compared to publicly available repositories used in previous studies. As an example, the close collaboration with the data collection team allowed for the resolution of several data provenance issues that typically arise during data analysis, such as inconsistencies across features or the detection of outliers, thereby maximizing the amount of usable data. Indeed, the literature indicates that potentially informative electronic public datasets are susceptible to inaccuracies (Vyas et al., 2021). In addition, to assess patients' cognitive profile, scores from individual neuropsychological tests were considered. Compared to aggregated scores extracted from short neuropsychological test batteries, this potentially allows for a finer-grained analysis of risk factors.

Our study identified several neuropsychological variables that predict significant declines in general cognitive functioning over time. Specifically, low baseline performance in visuo-spatial short-term working memory, assessed via the Corsi block-tapping test, long-term verbal memory skills measured by the BSRT, and executive function related to the inhibition of cognitive interference, evaluated through the SCWT, were all associated with deterioration at T3. The role of memory disorders as prodromal symptoms of AD has been well established (Amieva et al., 2008; Grober et al., 2008), therefore the presence of such impairments at disease onset might be considered a foregone conclusion. Cognitive assessment of visuo-spatial and verbal memory is routinely employed in clinical settings to evaluate degenerative diseases. The Corsi block-tapping test has been already recognized as a crucial test for the diagnosis of AD differentiating patients from controls at moderate stages (Guariglia, 2007). Additionally, the story recall test has been shown to predict progression to dementia in patients with MCI (Park et al., 2017), while the Stroop test is commonly used to differentiate healthy aging from early AD in elderly populations (Hutchison et al., 2010). Our results align with existing literature, which indicates that episodic memory and executive functions are the cognitive domains most susceptible to deterioration in patients with early AD (for a review see Twamley et al., 2006; Salmon, 2012; Mortamais et al., 2017).

Moreover, our results corroborate the significance of neuropsychological data in predicting global cognitive deterioration. Despite the heterogeneity of the variables studied, Zhu et al. (2016) emphasized the importance of executive (ADNI-EF) and memory (ADNI-MEM) components, while Fisher et al. (2019) reported the significance of cognitive trials based on immediate and delayed recall items belonging to the MMSE and to the ADAS battery. Dansson et al. (2021) identifies the ADAS and the TMT cognitive tests as strong predictors of cognitive decline. However, none of these studies reported the significance of the spatial memory test (Corsi block-tapping test), nor the verbal memory test for structured material (BSRT) or the ability of inhibiting cognitive interference (SCWT) in the prediction, as we have found. Nevertheless, we must highlight that there is a lack of uniformity in the neuropsychological data employed in the cited literature; for instance, Zhu et al. (2016) utilized cognitive scores as composite variables (e.g., ADNI-EF and MEM), while Fisher et al. (2019) and Dansson et al. (2021) relied on short neuropsychological batteries like the ADAS, which did not include the Corsi span or Stroop tests.

Our analysis indicates that the baseline assessment of cognitive functions can predict global cognitive decline over time, a conclusion supported also by other studies applying AI algorithms to clinical data from AD patients (Zhu et al., 2016; Fisher et al., 2019; Dansson et al., 2021). Conversely, the existing literature largely overlooks the association between spatial memory and cognitive deterioration, with few studies incorporating spatial memory assessments. Among the multiple studies considered in the reviews by Twamley et al. (2006) and Salmon (2012), only one included the Corsi span test, while Mortamais et al. (2017) cited only two studies featuring it. In light of our findings, we believe it would be important to include the span space score in baseline neuropsychological assessments for MCI patients with AD biomarkers, as it may enhance the prediction of cognitive progression.

Regarding clinical comorbidities, the present study highlighted the association between the presence of hypertension and cognitive decline, measured as a drop of the MMSE score. Conversely, an opposite pattern was found for hypercholesterolemia, which might serve as a protective factor. The presence of hypertension is consistently related with cognitive decline and increased risk of dementia (Tzourio et al., 1999; McGrath et al., 2017). However, few studies found an opposite pattern (Wysocki et al., 2012; Streit et al., 2019), suggesting that the association between blood pressure and brain cognitive functions is intricate and might be modulated by study-specific factors, such as study design, population characteristics, and the specific cognitive domains assessed (Iadecola et al., 2016; Sierra, 2020). Our dataset does not allow to inspect this issue in greater detail, thus further research will be required to clarify the role of hypertension in cognitive function among elderly individuals.

Elevated cholesterol levels are a major risk factor for cardiovascular disease, but their role in late-life cognitive function, dementia and cognitive decline is less clear. For example, Liu et al. (2021) found that long-term increases in higher total cholesterol and non-high density lipoprotein cholesterol levels were substantially associated with decreased risks of global cognitive and memory function decline. More precisely, when measured in late-life, elevated cholesterol levels show no association with a worsening of cognitive functions, or even an inverse relationship (van Vliet, 2012). Cholesterol, crucially, is an important component of nerve cell membranes and participates in the metabolic activities of nerve cells, it is essential for the formation and maturation of synapses and plays an important role in the regulation of signal transduction through its function as a component of the cell membrane (van Vliet, 2012). Furthermore, cholesterol stores a large amount of energy, which can provide sustained energy to the brain, which is the most energy-consuming organ of the body (Steiner, 2020). Therefore, the role of cholesterol in brain protection might be different from its role in cardiovascular diseases.

Lastly, the link between functional variables and cognitive decline in older adults has not yet been fully elucidated in the literature. Instrumental activities included in the IADLs questionnaire involve skills requiring the recruitment of multiple cognitive processes (e.g. houseworks, managing medications and finances, driving), and thus more complex than basic self-care activities measured by the ADLs questionnaires. Limited functionality in IADLs were found in literature to be predictive of dementia (Pérès et al., 2008), even in individuals with normal cognitive performance at baseline (Di Carlo et al., 2007). The SHAP analysis confirms that even a subtle decline in IADLs, restricted to a single instrumental activity (IADL < 1), might be predictive of significant worsening in cognitive abilities.

Despite these encouraging findings, it is important from a methodological viewpoint to make some considerations. First, while the sample size of the current study is sufficient to achieve robust and reproducible results, it might not ensure the generalizability of the model to external data. Additionally, as our dataset is derived from two Italian hospitals, it may not fully represent the broader AD population, potentially limiting the model's applicability to different clinical settings. Second, the filtering process adopted in this study, while effective in reducing the total number of features and inconsistencies, may have led to the exclusion of potentially informative variables due to strict thresholds for missing values, data variability, and class imbalance. This could limit the model's ability to capture subtle but clinically relevant patterns. Lastly, further analyses could be designed to try to enhance model performance, such as different data preprocessing techniques (different handling of missing values and normalization) or ensemble methods to combine predictions from multiple diverse machine learning algorithms.

## 5 Conclusions

Our findings highlight the clinical and cognitive variables assessed at baseline that contribute to the deterioration of overall cognitive function or serve as protective factors. Identifying contributing factors of cognitive decline along the AD continuum is essential for monitoring clinical progression and evaluating the efficacy of treatments to slow or preserve cognitive decline (GUIDANCE, 2018; Livingston et al., 2024). These results emphasize the importance of examining specific comorbidities, targeted cognitive domains, and impairments in instrumental activities of daily living (IADL), in addition to broader cognitive abilities, in both healthy and AD older adults. These insights are fundamental for tailoring pharmacological treatments with respect to comorbidities and for developing potential rehabilitation intervention focused on specific cognitive domains. Regarding limitations, further research is required to evaluate the generalizability of our model across different patient populations, despite the study's multicentric nature. Furthermore, inclusion of genetic and imaging data might have improved the performance of the model, at the expense of added complexity.

## Data Availability

The datasets presented in this article are not readily available because the informed consent obtained from participants did not include authorization for data sharing. Consequently, the supporting data cannot be made publicly available. Requests to access the datasets should be directed to mmoroni@fbk.eu.

## Group members of the NeuroArtP3 Network

Filippo Gerli (IRCCS Fondazione Don Carlo Gnocchi, Florence, Italy), Guido Pasquini (IRCCS Fondazione Don Carlo Gnocchi, Florence, Italy), Claudia Niccolai (IRCCS Fondazione Don Carlo Gnocchi, Florence, Italy), Matteo Betti (AOU Careggi, Florence, Italy), Emilio Portaccio (Dipartimento NEUROFARBA, Università degli Studi, Florence, Italy), Maria Pia Amato (Dipartimento NEUROFARBA, Università degli Studi, Florence, Italy; IRCCS Fondazione Don Carlo Gnocchi, Florence, Italy), Rossi Andrea (Neuroradiology Unit, IRCCS Istituto Giannina Gaslini, Genoa, Italy; Department of Health Sciences (DISSAL), University of Genoa, Genoa, Italy), Parodi Costanza (Neuroradiology Unit, IRCCS Istituto Giannina Gaslini, Genoa, Italy), Ramaglia Antonia (Neuroradiology Unit, IRCCS Istituto Giannina Gaslini, Genoa, Italy), Tortora Domenico (Neuroradiology Unit, IRCCS Istituto Giannina Gaslini, Genoa, Italy), Severino Mariasavina (Neuroradiology Unit, IRCCS Istituto Giannina Gaslini, Genoa, Italy), Andrea Falini (IRCCS Ospedale San Raffaele e Università Vita-Salute San Raffaele, Milan, Italy), Antonella Castellano (IRCCS Ospedale San Raffaele e Università Vita-Salute San Raffaele, Milan, Italy), Nicolò Pecco (IRCCS Ospedale San Raffaele e Università Vita-Salute San Raffaele, Milan, Italy), Paola Scifo (IRCCS Ospedale San Raffaele, Milan, Italy), Sonia Calloni (IRCCS Ospedale San Raffaele, Milan, Italy).
