# Supplementary material for: Neuropsychological and clinical variables associated with cognitive trajectories in patients with Alzheimer's disease
Source: Front Aging Neurosci. 2025 May 27;17:1565006. doi: 10.3389/fnagi.2025.1565006 (PMC12149119; doi:10.3389/fnagi.2025.1565006)
Supplement: Supplementary file 1 [file Data_Sheet_1.pdf]

Supplementary Material

Table S1. Description of variables included in the study.

Variable	Variable Type	Categories	Definition
**DEMOGRAPHIC**			
Age (years)	Numerical	-	-
Education (years)	Numerical	-	Level of education, measured in years of formal education.
Sex	Binary	1 (female)/0 (male)	-
Working position/occupation	Categorical	Elementary, medium-low, medium, medium-high, major	Elementary (worker entry-level), medium-low (worker with experience but no responsibility), medium (worker with experience and responsibility), medium-high (worker with high experience and high responsibility), major has a strategic role (e.g. manager).
**CLINICAL**			
Disease duration	Numerical	-	Years between diagnosis and baseline visit.
Family history of diseases	Categorical	None, Dementia, Parkinson, Multiple Sclerosis, Lateral Amyotrophic sclerosis, Cerebral tumors, Psychiatric pathology	Presence of relatives up to the 2nd degree with the considered pathologies.
Smoking	Categorical	Not present, previous, current	Smoking habits self-reported by the patient and/or caregivers during visit.
Alcohol	Categorical	Not present, previous, current	Alcohol abuse self-reported by the patient and or/caregivers during visit.
Hypertension	Binary	1 (presence)/ 0 (absence)	Presence or absence of hypertension diagnosed by a specialist of the Italian Public Health System (cardiovascular disorders guidelines Italian Ministry of Health https://www.salute.gov.it).

Continued on next page

Variable	Variable Type	Categories	Definition
Hypercholesterolemia	Binary	1 (presence)/0 (absence)	Presence or absence of hypercholesterolemia diagnosed by a specialist of the Italian Public Health System (cardiovascular disorders guidelines Italian Ministry of Health https://www.salute.gov.it).
Cardiopathy	Binary	1 (presence)/0 (absence)	Presence or absence of cardiopathy diagnosed by a specialist of the Italian Public Health System (cardiovascular disorders guidelines Italian Ministry of Health https://www.salute.gov.it).
Thyroidopathy	Binary	1 (presence)/0 (absence)	Presence or absence of thyroidopathy diagnosed by a specialist of the Italian Public Health System (endocrinology disorders guidelines Italian Ministry of Health https://www.salute.gov.it).
Head trauma	Binary	1 (presence)/0 (absence)	Presence or absence of head trauma diagnosed by the neurologist involved in the study (assessed by structured MRI instrumental exam).
Diabetes	Binary	1 (presence)/0 (absence)	Presence or absence of diabetes diagnosed by a specialist of the Italian Public Health System (cardiovascular disorders guidelines Italian Ministry of Health https://www.salute.gov.it).
Liver disease	Binary	1 (presence)/0 (absence)	Presence or absence of liver disease diagnosed by a specialist of the Italian Public Health system (gastroenterological disorders guidelines Italian Ministry of Health https://www.salute.gov.it).
Tumors	Binary	1 (presence)/0 (absence)	Presence or absence of tumors diagnosed by a specialist of the Italian Public Health system (oncology guidelines Italian Ministry of Health https://www.salute.gov.it).

Continued on next page

Variable	Variable Type	Categories	Definition
Cerebrovascular disease (Fazekas >1)	Binary	1 (presence)/0 (absence)	Substantial concomitant cerebrovascular disease, defined by a history of a stroke temporally related to the onset or worsening of cognitive impairment; or the presence of multiple or extensive infarcts or severe white matter hyperintensity burden.
CDR score	Categorical	0 (MCI)/0.5 (mild)/ 1 (moderate)	Severity of dementia at the Clinical Dementia Rating Scale (CDR).
Motor symptoms: Falls	Binary	1 (presence)/0 (absence)	Neurologic examination for motor symptoms.
Motor symptoms: Dysphagia	Binary	1 (presence)/0 (absence)	Neurologic examination for motor symptoms.
Motor symptoms: Parkinsonism	Binary	1 (presence)/0 (absence)	Neurologic examination for motor symptoms.
Motor symptoms: Others	Binary	1 (presence)/0 (absence)	Neurologic examination for motor symptoms.
Behavioral symptoms: Depression	Binary	1 (presence)/0 (absence)	Neuropsychiatric Inventory (NPI).
Behavioral symptoms: Apathy	Binary	1 (presence)/0 (absence)	Neuropsychiatric Inventory (NPI).
Behavioral symptoms: Hallucinations	Binary	1 (presence)/0 (absence)	Neuropsychiatric Inventory (NPI).
Behavioral symptoms: Delusions	Binary	1 (presence)/0 (absence)	Neuropsychiatric Inventory (NPI).
Behavioral symptoms: Aggression	Binary	1 (presence)/0 (absence)	Neuropsychiatric Inventory (NPI).
Behavioral symptoms: Disinhibition/Inadequate behavior	Binary	1 (presence)/0 (absence)	Neuropsychiatric Inventory (NPI).
Behavioral symptoms: Sleep disorders	Categorical	1 (presence)/0 (absence)	Neuropsychiatric Inventory (NPI).

Continued on next page

Variable	Variable Type	Categories	Definition
Pharmacological therapy	Categorical	None, cholinesterase inhibitors, memantine, benzodiazepines, antipsychotics, antidepressants, antidiabetics, antiplatelet agents, anticoagulants, antihypertensives, statins	-
**MEASURES OF FUNCTIONAL ABILITIES**			
ADLs	Numerical	-	ADL scores as a fraction between the number of preserved and total number of tested activities. According to this definition, ADL scores equal to 1 denote full independence, while values smaller than 1 denote impairment in some functionalities with smaller values denoting greater impairment.
IADLs	Numerical	-	IADL scores as a fraction between the number of preserved and total number of tested activities. [see ADLs definition]

Table S2. Evaluation of predictive model performance across different data splits. For each machine learning model (i.e., Logistic Regression, Random Forest, and Gradient Boosting), performance metrics for different test set proportions (i.e., 10%, 20%, 30%, and 40%) are reported as mean +/- sd.

Test Set %	Metric	Logistic Regression	Random Forest	Gradient Boost
10%	MCC	0.146 ± 0.294	0.234 ± 0.223	0.251 ± 0.201
	Balanced Accuracy	0.570 ± 0.145	0.615 ± 0.111	0.617 ± 0.094
	F1 Score	0.571 ± 0.166	0.630 ± 0.106	0.658 ± 0.116
	AUC	0.583 ± 0.175	0.640 ± 0.094	0.643 ± 0.152
	AUPRC	0.626 ± 0.176	0.649 ± 0.125	0.664 ± 0.159
20%	MCC	0.147 ± 0.294	0.232 ± 0.138	0.186 ± 0.100
	Balanced Accuracy	0.573 ± 0.091	0.615 ± 0.069	0.591 ± 0.051
	F1 Score	0.534 ± 0.207	0.632 ± 0.077	0.634 ± 0.056
	AUC	0.598 ± 0.111	0.658 ± 0.079	0.628 ± 0.086
	AUPRC	0.653 ± 0.111	0.699 ± 0.084	0.632 ± 0.075
30%	MCC	0.135 ± 0.103	0.215 ± 0.100	0.235 ± 0.122
	Balanced Accuracy	0.567 ± 0.051	0.607 ± 0.049	0.615 ± 0.060
	F1 Score	0.552 ± 0.191	0.631 ± 0.068	0.671 ± 0.063
	AUC	0.609 ± 0.091	0.643 ± 0.040	0.632 ± 0.075
	AUPRC	0.673 ± 0.082	0.694 ± 0.055	0.656 ± 0.068
40%	MCC	0.127 ± 0.124	0.170 ± 0.099	0.140 ± 0.076
	Balanced Accuracy	0.563 ± 0.062	0.584 ± 0.049	0.567 ± 0.036
	F1 Score	0.602 ± 0.058	0.614 ± 0.053	0.638 ± 0.043
	AUC	0.575 ± 0.060	0.625 ± 0.045	0.606 ± 0.052
	AUPRC	0.648 ± 0.063	0.678 ± 0.057	0.647 ± 0.065
